# Dosing Patterns during Conversion to IPX066, Extended-Release Carbidopa-Levodopa (ER CD-LD), in Parkinson's Disease with Motor Fluctuations

**DOI:** 10.1155/2018/9763057

**Published:** 2018-10-22

**Authors:** John C. Morgan, Rohit Dhall, Robert Rubens, Sarita Khanna, Suneel Gupta

**Affiliations:** ^1^Movement Disorders Program, Parkinson's Foundation Center of Excellence, Department of Neurology, Medical College of Georgia, Augusta University, 1429 Harper Street, HF-1154, Augusta, GA 30912, USA; ^2^University of Arkansas for Medical Science, 4301 W. Markham Street, Little Rock, AR 72205, USA; ^3^Impax Laboratories, LLC, 31047 Genstar Road, Hayward, CA 94544, USA

## Abstract

**Background:**

IPX066 is an extended-release (ER) oral formulation of carbidopa-levodopa (CD-LD). Following an initial peak at about one hour, plasma LD concentrations are maintained for about 4-5 hours.

**Objective:**

To present dosing factors that may affect the successful conversion to ER CD-LD from other LD formulations.

**Methods:**

Two-phase 3 studies of ER CD-LD vs. immediate-release (IR) CD-LD (ADVANCE-PD) and vs. CD-LD + entacapone (CLE; ASCEND-PD) in subjects with advanced PD included a 6-week, open-label conversion to ER CD-LD prior to treatment randomization. The “converted” daily LD dose ratio and dose frequency for ER CD-LD were compared to the prior LD treatment regimens at study entry.

**Results:**

The average daily LD dose ratio at the end of dose conversion to ER CD-LD was approximately 2.1 for IR CD-LD and 2.8 for CLE. The final dose ratios tended to be slightly higher for participants taking lower LD doses at study entry but independent of dose frequency. ER CD-LD dose frequency increased with increasing LD dose and dose frequency at study entry. Participants on higher baseline LD doses ≥800 mg and dose frequencies ≥6 tended to have higher rates of discontinuation during conversion to ER CD-LD.

**Conclusions:**

Converting participants from other LD formulations to ER CD-LD is based on their current LD regimen. For the most common daily doses (≤1250 mg) and dose frequencies (<7) of LD, final mean dose ratios were within tight ranges of 2.1 to 2.4 for IR CD-LD (ADVANCE-PD) and 2.4 to 2.8 for CLE (ASCEND-PD) and were generally independent of the LD dosing frequency at study entry. These trials are registered with NCT00974974, NCT01130493.

## 1. Introduction

The mainstay of pharmacological treatment for Parkinson's disease (PD) is the oral administration of levodopa (LD), coupled with an aromatic amino acid decarboxylase (AADC) inhibitor such as benserazide or carbidopa (CD). An AADC inhibitor reduces peripheral degradation of LD [1] and increases the amount of LD that ultimately reaches the brain [2]. Three times a day treatment with CD-LD is usually effective in patients with early PD [2, 3]. The plasma half-life of LD is only 1-2 hours [4, 5], however, and with time patients will often require more frequent administration of CD-LD during the day to maintain mobility [3, 6]. Other strategies to boost the duration of benefit from CD-LD include the addition of a catechol-O-methyl transferase (COMT) inhibitor, such as entacapone or tolcapone, or a selective monoamine oxidase type-B (MAO-B) inhibitor, such as selegiline or rasagiline, which reduces the rate of dopamine metabolism [7]. A controlled release CD-LD product is also available; however, its use has been limited by an increased time to onset of effect (participants often require some concomitant standard LD), increased dyskinesia, and a less reliable response particularly with postmorning doses compared to IR [8–10].

Extended-release (ER) CD-LD (IPX066, Rytary®, Numient^TM^) was developed to prolong therapeutic LD plasma levels in the treatment of PD and comes as a capsule formulation containing both immediate-release and extended-release beads of CD-LD. It is designed to achieve therapeutic LD plasma concentrations as quickly as IR CD-LD and to maintain them for a prolonged duration [11]. Previously published results of the randomized, double-blind, active comparator-controlled clinical trials have reported the efficacy and safety of ER CD-LD compared with IR CD-LD (ADVANCE-PD) [12] and with CD-LD plus entacapone (CLE, ASCEND-PD) [13] in advanced PD. In both of these studies, ER CD-LD significantly decreased daily “off” time and increased daily “on” time without troublesome dyskinesia vs. each of the active comparators.

Nausieda et al. [14] followed the primary ER CD-LD clinical trial data with a report describing the dose conversion periods of each study in more detail, when participants were converted from their previous CD-LD formulation (IR or CLE) to ER CD-LD. The majority of participants (87.3% and 82.7%, respectively) who entered the dose conversion phase were able to be successfully converted to ER CD-LD and enter the double-blind portion of each clinical trial. However, since the LD exposure after a single dose of ER CD-LD (as measured by the area under the time curve of LD plasma level) averaged approximately 70% and the peak plasma LD concentration (*C*_max_) averaged approximately 30% of the values following a single dose of IR CD-LD [15, 16], changes in LD dose and dose frequency were expected when participants were converted to ER CD-LD. In line with this prediction, in the overall study populations, the average ratio of daily LD dose (mg/day) from ER CD-LD was approximately 2.1 after conversion from IR CD-LD and approximately 2.8 after conversion from CLE. Additionally, the required dosing frequency decreased from a mean of 5.1 and 5.0 times per day for IR CD-LD and CLE to 3.6 and 3.5 times per day for ER CD-LD in the respective studies.

The intent of this manuscript is to provide physicians with additional detail to help initiate conversion to ER CD-LD based on previous dosing regimens.

## 2. Methods

### 2.1. Study Designs

Study designs for both ADVANCE-PD and ASCEND-PD have been published previously [12, 13]. Briefly, ADVANCE-PD was a randomized, double-blind, double-dummy, parallel-group study of ER CD-LD versus IR CD-LD. Prior to randomization and 13-week double-blind treatment, the prestudy IR CD-LD regimen was optimized over 3 weeks for each participant based on clinical response. Open-label conversion to ER CD-LD was conducted during the subsequent 6 weeks. In ASCEND-PD, a randomized, double-blind, double-dummy, crossover study of ER CD-LD versus CLE, participants were required to be on a stable CLE regimen for at least 4 weeks at study entry, and CLE dosing was not adjusted on-study. Similar to ADVANCE-PD, conversion to ER CD-LD was conducted open-label over a 6-week period. This was followed by two 2-week, double-blind crossover treatment periods. The double-blind treatment periods were separated by a week of open-label treatment with ER CD-LD. The use of amantadine, anticholinergics, selective MAO type B inhibitors, or dopamine agonists at stable dosages (at least 4 weeks before screening) was allowed in both studies; however, participants were not permitted to initiate such agents or use any nonstudy LD preparations.

### 2.2. Study Participants

Participants had a diagnosis of idiopathic PD by United Kingdom Parkinson's Disease Brain Bank Criteria [17], were ≥30 years of age, and had Hoehn and Yahr [18] stages 1 to 4 during an “on” state. All participants were also required to have at least 2.5 hours/day of “off” time, in spite of a stable regimen of oral CD-LD for at least 4 weeks prior to screening, taken at least 4 times/day and totaling at least 400 mg/day of LD from either IR CD-LD (ADVANCE-PD) or CLE (ASCEND-PD).

### 2.3. Conversion to IPX066

To help identify an appropriate initial ER CD-LD regimen, study investigators were given conversion tables based on the prestudy LD daily dosages, ranging from a minimum of 400 mg/day to >1650 mg/day of LD when converting from IR CD-LD and to >1250 mg/day when converting from CLE [14]. Study teams were provided guidance for conversion from the current LD regimen to ER CD-LD. Because entacapone increases AUC by approximately 30% over CD-LD alone, these participants received higher doses than those on CD-LD alone (see Table 1 (ADVANCE-PD) and Table 2 (ASCEND-PD)). Dosing frequencies were initiated at three times per day, but adjustment to 4 or 5 times per day was allowed to improve total “on” time if less frequent dosing did not accomplish enough “on” time.

### 2.4. Conversion-Data Analyses

For participants successfully transitioning to ER CD-LD, mean conversion ratios (ER CD-LD daily mg per day/prior daily LD mg per day) and mean ER CD-LD dose frequencies are summarized by LD IR or CLE dose and dosing frequency at study entry [14]. The rates of participant discontinuation during conversion to ER CD-LD were summarized by baseline LD dose and dosing frequency at study entry to examine baseline dosing characteristics that may have resulted in higher or lower rates of discontinuation during conversion.

### 2.5. Concomitant Medications

A post hoc analysis of data from both studies was completed to evaluate whether the efficacy and safety of ER CD-LD relative to the respective active comparators were affected by concomitant medications. In each study, the randomized patient population was divided into 3 subgroups based on the use of concomitant medications at study entry: a dopaminergic agonist group, a MAO-B inhibitor group (rasagiline and selegiline), and an amantadine group. If patients could be receiving more than 1 of these adjunctive medications concurrently, they were included in more than 1 subgroup for analysis. For each subgroup, the changes from baseline in PD diary measures (“off” time and “on” time with and without troublesome dyskinesia) and UPDRS sum of Parts II (activities of daily living) and III (motor examination) scores in the “on” state were analyzed.

### 2.6. Ethical Conduct

Both studies were conducted in accordance with Good Clinical Practice guidelines, and study procedures were approved by appropriate institutional review boards. All participants provided written informed consent prior to taking part in any study activities [12–14].

## 3. Results

Participant disposition for the overall studies and numbers of successful conversion to ER CD-LD have been published previously [12–14]. Briefly, in the ADVANCE-PD study for patients who were randomized (*n*=393), the mean age was 63.2 years, and 65% were male, the mean PD duration was 7.4 years and the mean baseline “off” time was 5.97 hours [12]. In the ASCEND-PD study, patients (*n*=91) were randomized; the corresponding baseline demographics for these patients were 64.1 years, 75% males, mean PD duration of 10.0 years, and mean baseline “off” time of 5.9 hours [13].

### 3.1. Final ER CD-LD Conversion Ratios and Dose Frequency by Baseline LD Dose

**Figure 1:**
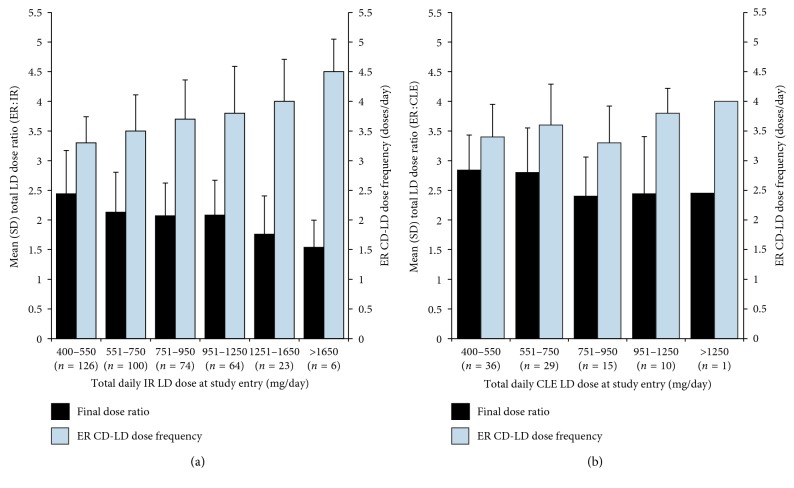
Final daily LD dose ratios (black bars) and ER CD-LD daily dosing frequency (gray bars) based on daily LD dose ranges at study entry for the conversion from IR CD-LD (a) or CLE (b).

Figure 1 shows final daily mean ER : IR LD dose ratios (black bars) and ER dose frequencies (gray bars) after conversion to ER CD-LD for each daily IR LD dose (mg/day) at study entry. Panel A highlights that participants who entered the trial taking lower IR CD-LD doses (400–550 mg/day of LD) tended to have LD dose ratios >2 (requiring a relatively higher daily LD dose from ER CD-LD), whereas participants with higher daily IR LD doses (>1250 mg/day) tended to have final dose ratios <2 (requiring a relatively lower daily LD dose from ER CD-LD). A similar trend was seen for participants taking lower CLE doses at study entry, although the conversion ratios were higher (2.4–2.8) when converting from CLE and only one participant was included who was taking >1250 mg/day of CLE (Figure 1(b)). These small ratio trends notwithstanding, for the most common daily doses (≤1250 mg) and dose frequencies (<7) of LD, final mean dose ratios were within a tight range of 2.1 to 2.4 for IR CD-LD (ADVANCE-PD) and 2.4 to 2.8 for CLE (ASCEND-PD).

The final dose frequencies for ER CD-LD demonstrate that the vast majority of participants, those on 400–1650 mg/day of IR CD-LD or 400–1250 mg/day of CLE at study entry, tended to convert to ER CD-LD on a TID or QID dosing regimen (Figures 1(a) and 1(b), gray bars).

### 3.2. Final ER CD-LD Conversion Ratios and Dose Frequency by Baseline LD Dose Frequency

**Figure 2:**
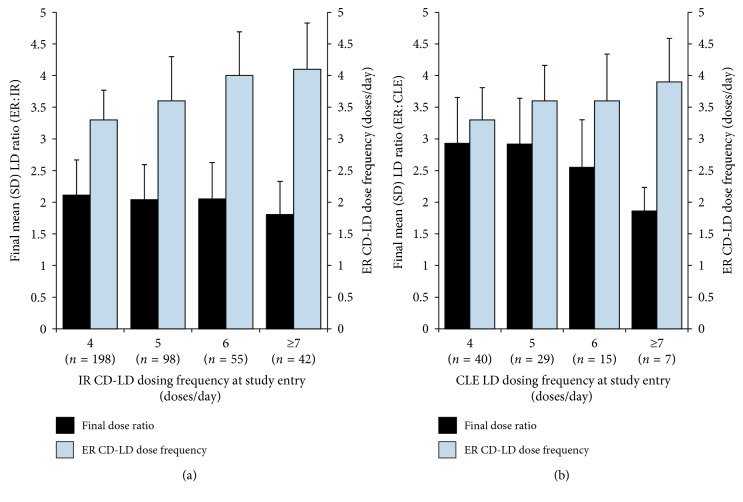
Final daily LD dose ratios (black bars) and ER CD-LD daily dosing frequency (gray bars) based on daily LD dosing frequency at study entry for the conversion from IR CD-LD (a) or CLE (b).

Figure 2 shows the final daily ER : IR LD dose ratios (black bars) and ER dose frequencies (gray bars) after conversion to ER CD-LD, based on the daily dose frequency of IR (Panel A) or CLE (Panel B) at study entry. The final conversion ratio of ER CD-LD to IR CD-LD or CLE was generally independent of the frequency of LD dosing at study entry; however, there was a tendency for participants taking LD 7 or more times per day at baseline to have a slightly lower conversion ratio (1.8), indicating that a relatively lower daily ER CD-LD dose was required.

The final dose frequencies for ER CD-LD tended to increase between approximately 3.3 and 4.1 with increased LD dosing frequency at baseline (Figures 2(a) and 2(b), gray bars). Participants taking IR CD-LD or CLE 4 times/day were more likely to convert to ER CD-LD on a TID regimen; however, as baseline LD dose frequency increased to 6 or more times/day with IR CD-LD and to 7 or more times/day with CLE, the frequency of ER CD-LD dosing reached a mean of approximately 4 times/day.

### 3.3. Discontinuations by Baseline LD Dose

**Figure 3:**
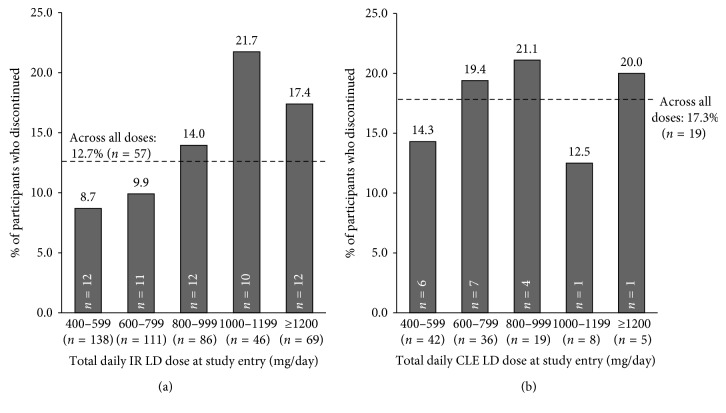
Proportions of participants who discontinued during dose conversion based on daily LD dose ranges at study entry during the conversion from IR CD-LD (a) or CLE (b). Proportions were calculated using the total number of participants who entered dose conversion (*n*=450 in (a) *n*=110 in (b)). The numbers of participants who discontinued from each dose range are indicated within each bar. The dashed lines represent the overall rate of discontinuation during dose conversion in each study.

**Figure 4:**
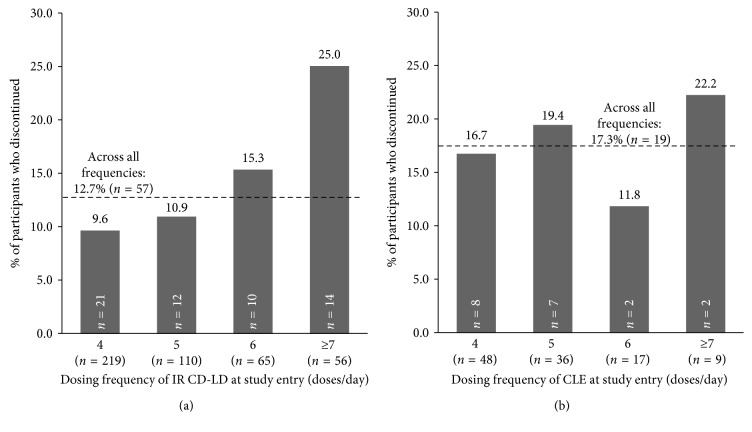
Proportions of participants who discontinued during dose conversion based on daily LD dose frequency at study entry during the conversion from IR CD-LD (a) or CLE (b). Proportions were calculated using the total number of participants who entered dose conversion (*n*=450 in (a) *n*=110 in (b)). The numbers of participants who discontinued from each dose range are indicated within each bar. The dashed lines represent the overall rate of discontinuation during dose conversion in each study.

The rates of participant discontinuation during dose conversion by baseline LD daily dose and dosing frequency are shown in Figures 3 and 4. The overall rate of discontinuation during conversion to ER CD-LD was 12.7% (*n*=57) in ADVANCE PD and 17.3% (*n*=19) during ASCEND-PD [12–14]. Participants on lower daily LD doses and dosing frequencies at baseline tended to have lower rates of discontinuation in both studies, and participants on higher daily LD doses and dosing frequencies tended to discontinue more frequently.

### 3.4. Concomitant Medications

Of the patients randomized, at least 50% in both studies received a concomitant dopaminergic agonist; however, fewer than 25% of patients took amantadine in either trial. Fewer than 25% of patients took selegiline or rasagiline in ADVANCE-PD, whereas 35% of patients took either one of these in ASCEND-PD. The full results including efficacy and safety analyses have been reported [15].

Pertinent to dose conversion, the final dose ratios of pre- and postconversion to ER CD-LD from IR CD-LD were all 2.0-2.1 regardless of the concomitant medication subgroup. For conversion to ER CD-LD from CLE, the ratio was 2.7–2.9 for the concomitant medication subgroups except for the amantadine group where the ratio was 2.5 [15].

In the ADVANCE-PD trial, the mean number of discontinuations between participants who were not being treated with one of these concomitant medications and with those participants in each of the concomitant medication subgroups was similar. There were only 4 early discontinuations in the ASCEND-PD study overall (all in the ER CD-LD subgroup), so no pattern could be discerned.

The proportions of patients taking each class of medication were similar between participants who completed conversion and those who did not. During conversion from IR CD-LD (ADVANCE-PD), 23 patients (5.1%) discontinued due to adverse events (AEs). The AEs leading to discontinuation of more than one patient were dyskinesia (in 5 patients, or 1.1%); anxiety, dizziness, and “on” and “off” phenomenon (each in 3 patients, or 0.7%); and nausea and visual hallucinations (each in 2 patients, or 0.4%). During conversion from CLE (ASCEND-PD), one patient (0.9%) discontinued due to dyspepsia, nausea, and vomiting [14]. The overall safety profile of ER CD-LD was not altered by the use of these concomitant medications.

## 4. Discussion

ADVANCE-PD and ASCEND-PD demonstrated that participants treated with ER CD-LD benefitted from a reduced mean daily “off” time and increased mean daily “on” time without troublesome dyskinesia compared to both IR [12] and CLE [13]. Two retrospective chart reviews of ER CD-LD used within individual movement disorder clinics have reported lower rates of successful conversion of participants to ER CD-LD [19, 20] than the 87% successful conversion rate reported in the ADVANCE-PD study, suggesting the need for further guidance for successful conversion. The current analyses should provide additional information for physicians who are considering ER CD-LD for their participants, as they demonstrate that the final mean conversion dose ratios for the most common daily doses (≤1250 mg) and dose frequencies (<7) of LD were within tight ranges of 2.1 to 2.4 for IR CD-LD (ADVANCE-PD) and 2.4 to 2.8 for CLE (ASCEND-PD) and were generally independent of the LD dosing frequency at study entry. Additionally, the analysis of the relationship between participant dosing regimens and the rate of discontinuations during conversion in the clinical trials may help identify participants for whom conversion may be facilitated by more frequent follow-up or monitoring.

On average, participants who successfully converted to ER CD-LD needed approximately twice the daily LD dose in ER CD-LD that was in their IR CD-LD regimen at study entry [12, 14] and approximately 2.8X the daily LD dose in ER CD-LD relative to the IR LD dose in their CLE [13, 14]. As an example, a participant on one tablet of IR CD-LD 25/100 mg six times per day (total of 600 mg of IR LD/day) would likely need to be converted to approximately 1200 mg of ER CD-LD divided into four daily doses. A reasonable approximation would be two 36.25/145 mg capsules QID, for a total daily dose of 1160 mg LD in ER CD-LD. However, the current analyses extended the previous work to show that participants who were on lower daily doses of LD tended to need a slightly higher daily ER : IR LD dose ratio. So, while a physician may decide to start the participant closer to 2X the dose, the expectation may be that the dose of ER CD-LD will need to be increased to obtain an adequate clinical response. Conversely, participants on LD daily doses higher than 1250 mg dose frequencies at study entry tended to require proportionally lower ER : IR LD dose ratio, and hence, more advanced participants taking higher doses of LD may be expected to have a better response from a daily dose of ER CD-LD that is below the 2X threshold. As stated above, these final conversion dose ratios are relatively independent of the baseline IR LD dosing frequency.

The increase in daily LD dosage and decreases in dosing frequency with ER CD-LD are reflective of the pharmacokinetic differences of ER CD-LD vs. IR CD-LD and CLE [16]. The bioavailability of LD from ER CD-LD is approximately 70% of that from an equivalent dose of IR CD-LD [11]. Additionally, Hauser et al. [11] reported that plasma LD levels in advanced PD participants remained above 50% of *C*_max_ for a mean (SD) of 4.0 (2.0) hours after a single dose of ER CD-LD, compared with 1.4 (0.7) hours for IR CD-LD. Because of its ability to sustain therapeutic LD plasma concentrations, ER CD-LD acts to “fill in” the troughs in LD concentration between doses of standard oral LD formulations, and hence, it necessitates an increase in LD per individual dose.

The examination of discontinuations also provides a means for setting expectations regarding successful conversion. The results of conversion from IR CD-LD indicate that participants on lower doses and dose frequencies of LD at study entry also had lower rates of discontinuation than those on higher doses and dose frequencies. A similar trend was seen for participants converting from CLE; however, the lower number of participants in the ASCEND-PD trial may have contributed to some of the variability in the CLE results. Lower doses/dosing frequencies of LD likely represent surrogate markers of less advanced disease; it makes sense that patients with more advanced disease will likely require more intensive medical attention during conversion.

It is important to acknowledge that the successful treatment of PD requires good communication of potential risks and benefits of any therapeutic intervention. The data from ADVANCE-PD and ASCEND-PD suggest that many patients will see improvement in “off” time without an increase in troublesome dyskinesia with a final ER LD regimen that is approximately double the current CD-LD or CLE dosage, when given three to four times daily. However, the conversion to a new levodopa therapy, such as ER CD-LD, requires a careful management of expectations. Providing clear dosing titration instructions to patients and caregivers may facilitate conversion and optimize the patient's “good on” time [21].

## Figures and Tables

**Table 1:** ADVANCE-PD dose conversion from IR CD-LD to ER CD-LD

Total daily dose of levodopa in immediate-release carbidopa-levodopa	Recommended staring dosage of RYTARY	
	Total daily dose of levodopa in RYTARY	RYTARY dosing regimen
400 mg to 549 mg	855 mg	3 capsules RYTARY 23.75 mg/95 mg taken TID^a^
550 mg to 749 mg	1140 mg	4 capsules RYTARY 23.75 mg/95 mg taken TID^a^
750 mg to 949 mg	1305 mg	3 capsules RYTARY 36.25 mg/145 mg taken TID^a^
950 mg to 1249 mg	1755 mg	3 capsules RYTARY 48.75 mg/195 mg taken TID^a^
Equal to or greater than 1250 mg	2340 mg or 2205 mg	4 capsules RYTARY 48.75 mg/195 mg taken TID^a^ or 3 capsules RYTARY 61.25 mg/245 mg taken TID^a^

^a^TID: three times a day.

**Table 2:** ASCEND-PD dose conversion from IR CD-LD + entacapone to ER CD-LD

Total daily dose of levodopa in immediate-release carbidopa-levodopa + entacapone	Recommended staring dosage of RYTARY	
	Total daily dose of levodopa in RYTARY	RYTARY dosing regimen
400–500 mg	1140 mg	380 mg (4 capsules × 95 mg) taken TID^a^
551–750 mg	1470 mg	490 mg (2 capsules × 245 mg) taken TID^a^
751–950 mg	1755 mg	585 mg (3 capsules × 195 mg) taken TID^a^
951–1250 mg	2205 mg	735 mg (3 capsules × 245 mg) taken TID^a^
>1250 mg	2940 mg	980 mg (4 capsules × 245 mg) taken TID^a^

^a^At 6-hour intervals during the subject's waking day; TID: three times a day.

## Data Availability

The underlying data and other supportive data have been published previously and are referenced throughout the manuscript.
